# Validity and Reliability of the Multidimensional Scale of Perceived Social Support (MSPSS) in Greek Secondary School Students

**DOI:** 10.3390/children12060706

**Published:** 2025-05-29

**Authors:** Vasiliki Efthymiou, George P. Chrousos, Kalliopi Kounenou, Antonios Kalamatianos, Christos Pezirkianidis, Markos Korelopoulos, Charikleia Stefanaki, Ntina Kourmousi

**Affiliations:** 1 Center for Adolescent Medicine and UNESCO Chair in Adolescent Health Care, First Department of Pediatrics, Medical School, National and Kapodistrian University of Athens, Aghia Sophia Children’s Hospital, 11527 Athens, Greece; chrousge@med.uoa.gr (G.P.C.); cstefanaki@gmail.com (C.S.); 2 University Research Institute of Maternal and Child Health and Precision Medicine, National and Kapodistrian University of Athens, 11527 Athens, Greece; 3 Department of Education, School of Pedagogical & Technological Education, 15122 Marousi, Greece; kkounen@aspete.gr (K.K.); nkourmousi@aspete.gr (N.K.); 4 Department of Education, School of Education, University of Nicosia, Nicosia 2417, Cyprus; kalamatianos.a@unic.ac.cy; 5 Laboratory of Positive Psychology, Panteion University of Social & Political Sciences, 17671 Athens, Greece; pezir@panteion.gr; 6 Ministry of Education, Experimental School of University of Athens, 10673 Athens, Greece; mscedt21040@uniwa.gr

**Keywords:** social support, adolescence, family, significant others, friends

## Abstract

**Background/Objectives**: Resilience—a complex phenomenon embracing a number of factors on various levels—seems to be a most important skill to survive this vulnerable phase. One major factor is the perceived social support during adolescence. Recognizing gaps in perceived social support may lead to prevention of serious social and medical problems, including juvenile delinquency and stress-related diseases. Screening questionnaires to identify the gaps in perceived social support in Greek adolescents are lacking. The purpose of this study was to validate a Greek version of the Multidimensional Scale of Perceived Social Support (MSPSS), a self-report scale of perceived social support of the family, significant others, and friends. **Methods**: The MSPSS was translated into Greek and was administered to students of 15 to 18 years of age from several areas of Greece. Psychometric properties of the MSPSS tool were investigated by Exploratory Factor Analysis (EFA), using principal component analysis with the Varimax Rotation Method. For Confirmatory Factor Analysis (CFA), the Comparative Fit Index (CFI), Tucker–Lewis index (TLI), and the Root Mean Square Error of Approximation (RMSEA) were used. **Results**: A total of 999 students completed the MSPSS anonymously. The Greek version of the MSPSS scale exhibited large internal consistency (Cronbach’s α of 0.932, McDonald’s Omega of 0.926). Three of the factors explained 80.80% of total variance. **Conclusions**: The Greek adaptation of the MSPSS is a valid instrument, and professionals can apply it as a screening tool for perceived social support in adolescents.

## 1. Introduction

Adolescence is a delicate and life-transition period of youth. Resilience is recognized as a crucial skill for managing this delicate period, encompassing a multifaceted concept that integrates numerous factors across different dimensions. One major factor is the perceived social support during adolescence. Recognizing gaps in perceived social support may lead to prevention of serious social and medical problems, including juvenile delinquency and stress-related diseases. Stress levels of the contemporary lifestyle are amplified, having detrimental effects on the health levels, well-being, and mental health of adolescent high-schoolers [1]. On the other hand, a large number of studies suggest social support among high school students as a vital factor influencing their mental and physical health, academic performance, coping strategies, and overall well-being. Strong social support networks during adolescence play an important role in shaping emotional, social, and academic development, contributing to a healthier and more fulfilling life.

In general, social support refers to the emotional, informational, and practical assistance received by individuals via their social networking, including family, friends, colleagues, and community members [2,3,4]. Social support seems to play a crucial role in promoting mental and physical well-being, helping individuals cope with stress, and fostering resilience in challenging times [5,6,7]. There are several types of social support: (i) Emotional Support: providing empathy, care, love, and trust. It helps individuals feel understood and valued [8,9]; (ii) Informational Support: such as advice, guidance, and information, helping individuals make decisions or solve problems [10]; (iii) Instrumental Support: tangible assistance, such as financial help, services, or physical aid during times of need [11,12]. Social support is of ultimate importance, as it seems to reduce feelings of isolation, enhance self-esteem, and promote a sense of belonging [13].

Traditional Greek culture accentuates strong family bonding, ties, and community relationships, which consist of the types of social support available [14]. Cultural context assesses the interrelationships among these cultural values, perceptions of support, and coping strategies [15,16]. Research on social support has gained attention in recent years, exploring its impact on mental health, community resilience, and well-being. Researchers have investigated the impact of social support in alleviating mental health challenges, including depression and anxiety, especially during economic crises or natural disasters [6,17,18,19]. Findings imply robust social networks can serve as protective factors against mental health issues that stress imposes [20,21]. Concerning Greece, its financial crisis led to increased interest in the social support buffering of the effects of economic stress and in the role of community support initiatives in helping individuals cope with unemployment and financial hardship [22,23,24]. Also, social support has been found to positively influence physical health, promote healthier lifestyles, and improve recovery from illnesses [8]. Recent data revealed that social support affects youth in a prominent way, particularly in terms of academic performance, social integration, and mental health [25,26,27]. Overall, in Greece, there are but a few studies that have assessed the value and significance of social support and highlighted the interplay between cultural norms, community structures, and individual well-being, and provided valuable insights into the impact of support systems on the enhancement of improved health outcomes [28,29].

There are several tools and scales for the assessment of social support, each varying in main outcome and methodology. The Social Support Questionnaire (SSQ) assesses the perceived availability of social support and the satisfaction of the support [30]. The Interpersonal Support Evaluation List (ISEL) assesses the availability of social resources and includes scales such as tangible support, appraisal support, and belonging [31], and it has been validated in Greek university students [32]. The Social Support Inventory (SSI) measures different types of support, including emotional, informational, and tangible support, as well as the overall satisfaction with the support received [33]. The ISEL can also be administrated to adolescents, as well as the Child and Adolescent Social Support Scale (CASSS), which is a screening and research self-report tool that can be used to assess the social support perceived by children and adolescents [34]. Each of these tools has its strengths and limitations so researchers can choose based on the specific research question or clinical need. It is also crucial to consider the context, the population, and the psychometric characteristics, to ensure the optimum for the study’s outcome.

Comparisons between psychometric properties of the Multidimensional Scale of Perceived Social Support (MSPSS) have revealed its sufficient reliability and validity across various populations and cultures [35]. Various validation studies and cultural adaptations of the MSPSS have been published, in different target populations. Greek scales of the MSPSS have been validated in different populations, such as the general adult population [36], nurses [37], and patients diagnosed with multiple sclerosis [38]. Although the MSPSS has been validated across adolescent populations internationally, there is currently no published psychometric validation specifically targeting Greek secondary school students. While the MSPSS has been translated into Greek and applied in studies involving adults and university students, its psychometric properties have not been formally assessed in younger school-age populations. Given the unique cultural and educational characteristics of the Greek context, the present study aims to fill this gap by providing the first targeted validation of the MSPSS in a representative sample of Greek secondary school students, assessing its reliability and construct validity within this specific group.

Moreover, due to its multiple and expanded usage in studies across the world, and since the MSPSS is a brief and effective tool for screening perceived social support, it is valuable in research, but also in the clinical practice of a vulnerable population, such as adolescent high schoolers. Perceived social support from different sources has been proven to be beneficial for the well-being and quality of life of adolescents. It serves as a protective factor against internalizing symptoms in adolescents—such as depression, anxiety, and loneliness—while also enhancing positive emotional feelings like hope, well-being, and a sense of security. Also, many studies have found a positive association between perceived social support and adolescents’ life satisfaction. This study has as its main aim to validate the MSPSS as a tool assessing the perceived social support from family, friends, and significant others in adolescents [35] and to explore how various sources of social support relate to adolescents’ overall life satisfaction.

## 2. Materials and Methods

This validation study recruited 999 upper secondary school (lyceum) students from all Greek regions, from the 1st of March until the 30th of September 2023. To assess the adequacy of the sample size for factor analysis, we followed established guidelines suggesting a subject-to-item ratio of at least 5:1 to 10:1 [39,40]. With 12 items in the MSPSS and a sample of 999 participants, our study exceeds the minimum recommended threshold, supporting the stability of the factor structure. The research was organized by the School of Pedagogical and Technological Education (ASPETE—A.Σ.ΠAΙ.Τ.Ε.), a Greek University that specializes in training secondary school teachers, in collaboration with the UNESCO Chair on Adolescent Health Care. It was approved by the Ethics and Conduct Committee of the School of Pedagogical and Technological Education.

The inclusion criteria were to be a high school student in a Greek school of 15 to 18 years of age, attending any high school class, from the first, second, or third class, with the ability to understand, read, and write in Greek. Exclusion criteria included individuals who did not attend a Greek high school. The Multidimensional Scale of Perceived Social Support (MSPSS) was provided along with an embedded consent form.

The participants were recruited via an open online invitation of the School of Pedagogical and Technological Education (ASPETE—A.Σ.ΠAΙ.Τ.Ε.) to the parents of upper secondary students of Greek lyceums. The online invitation explained the scope of the study, as well as the ensuring of anonymity, while providing a link for the study questionnaire.

The MSPSS is a psychological tool designed to measure an individual’s perception of social support [36]. The MSPSS assesses the perceived degree of support of individuals of the general population in various domains of their life. The MSPSS is a useful tool for studies investigating the role of social support in terms of psychological well-being and coping mechanisms across different life stages and cultures. It has, also, been used in clinical trials for the assessment of the patients’ social support network and the identification of gaps of support [41].

It consists of 12 items, graded in a 7-point Likert scale ranging from 1 (Very Strongly Disagree) to 7 (Very Strongly Agree). The items fall by 4 into the scale’s 3 dimensions, which form its subscales:

- Family Support: Evaluates the perceived emotional and practical support a person feels they receive from their family.
- Friend Support: Assesses the degree of the perceived support from their friends.
- Significant Other Support: Focuses on perceived support from a special person or partner.

Higher scores in each subscale indicate a greater perception of support from the respective sources.

The MSPSS was translated into the Greek language from the original text, following standard translation protocols [42,43]. Two professional translators, who were native speakers of the Greek language (i.e., target) but also fluent in the English language (i.e., source) conducted independent forward translations into the target language, thus developing a preliminary Greek version, which was translated back into the original language by another professional translator. The original and back-translated versions of the scale were compared, and adjustments were resolved through appropriate modifications. The final version of the instrument was subsequently reviewed by an expert panel, who provided their feedback and suggestions for refinement. The produced scale was then administered to a small group of students aged 15–18, in order to further investigate the questionnaire items’ clarity and comprehension. After this process, the final Greek version of the MSPSS described below was produced.

The statistically significant level was set at 0.05. All statistical analyses were conducted via IBM© SPSS© version 29 statistical software (IBM Corp. Released 2023. IBM SPSS Statistics for Windows, Version 29.0.2.0 Armonk, NY, USA: IBM Corp) and R Statistics software version 4.0.3 [44] (R Core Team, 2020) using the Lavaan package [45] and LavaanPlot package [46]. Exploratory Factor Analysis (EFA) was assessed using Principal Component Analysis with Varimax Rotation Method. For Confirmatory Factor Analysis (CFA), the comparative fit index (CFI), Tucker–Lewis Index (TLI), and the root mean square error of approximation (RMSEA) were used. Internal consistency was evaluated using Cronbach’s alpha coefficient [47].

## 3. Results

### 3.1. Demographic Characteristics of the Sample

After the evaluation of inclusion and exclusion criteria, a total of 999 participants were included in this validation study (Table 1). The sample consisted of female (663 subjects; 66.4%), and male adolescents (331 participants, 33.1%); three adolescents identified as “other gender” (0.5%). The majority of the participants, 508 students (50.9%), derived from the 3rd grade of the upper secondary schools in Greece (lyceums), while 349 students (34.9%) derived from the second grade, and 14.2% derived from the first grade. Almost 9 out of 10 students declared they attended general secondary education (general lyceums), whereas the rest of the participants attended different types such as vocational, model, experimental, and art lyceums.

### 3.2. Exploratory Factor Analysis

Exploratory Factor Analysis (EFA) was assessed using principal component extraction method (Table 2). The Kaiser–Meyer–Olkin Measure (KMO) of Sampling Adequacy was computed as 0.920 (marvelous) [48], and Bartlett’s Test of Sphericity was statistically significant (*p* < 0.001). All items of the MSPSS had acceptable Measures of Sampling Adequacy (MSA) over the value of 0.5 with a range from 0.896 to 0.955. The mean of all items varied from 4.90 to 5.98.

**Figure 1:**
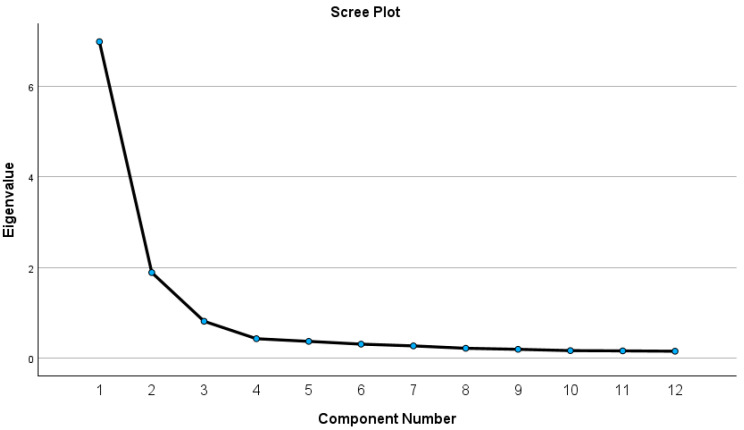
Scree plot.

Three components were extracted after Varimax rotation (Figure 1), while these dimensions had eigenvalues over 1 (3.48, 3.38, and 2.82, respectively), and the explained variances were 29.03%, 28.20%, and 23.57%, respectively (cumulative explained variance after rotation was equal to 80.80%). These three dimensions identified as the original MSPSS were ‘Significant Other’ (items 1, 2, 5, 10), ‘Family’ (items 3, 4, 8, 11) and ‘Friends’ (items 6, 7, 9, 12).

The full scale exhibited excellent internal consistency (Cronbach’s α = 0.932; McDonald’s ω = 0.926). Cronbach’s alpha for each scale was found to be 0.899 for ‘Significant Other’, 0.913 for Family, and 0.937 for Friends. McDonald’s Omega for each scale was found to be 0.899, 0.915, and 0.938 for each subscale, respectively.

### 3.3. Confirmatory Factor Analysis

**Figure 2:**
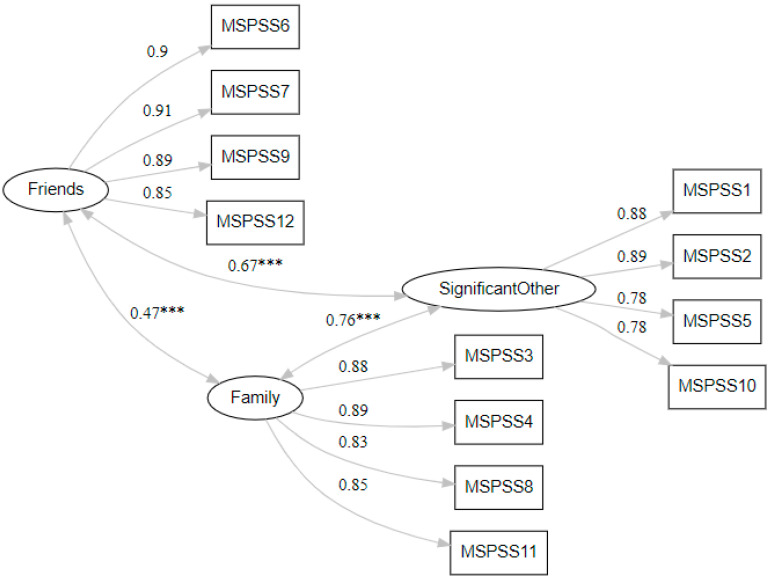
Confirmatory factor analysis for the Greek version of the “Multidimensional Scale of Perceived Social Support” (MSPSS). *** indicates p<0.001.

CFA was assessed in the sample of adolescents (Figure 2). Factor loadings indicated an adequate confirmation for the three-factor model of the MSPSS as recommended by the researchers of the original tool, while all standardized factor loadings were above 0.70, ranging from 0.78 to 0.91 (Figure 2). Statistically significant covariances between the three subscales were also revealed (*p* < 0.001). The strongest association emerged between Family and Significant Others (*r* = 0.76), followed by Friends and Significant Others (*r* = 0.67), while Friends and Family dimensions had the weakest association (*r* = 0.47). The estimation of factor loadings ranged from 1.12 to 1.57.

The goodness of fit indices also established that the investigated three-factor model was an adequate representation of the latent structure. The RMSEA (90% Confidence Interval—CI) was found equal to 0.08 (0.07–0.09); CFI was equal to 0.97; and TLI was equal to 0.96. The following cutoff points were used to evaluate good model fit: (i) an RMSEA in the range of 0.05 to 0.10 was considered an indication of acceptable fit, with values above 0.10 indicating poor fit [49] and values as high as 0.08 indicating an acceptable fit [50]; (ii) a value of CFI ≥ 0.95 is accepted as indicative of good fit [51]; (iii) TLI ≥ 0.95 is a cutoff criterion that is frequently used for the goodness of fit [52].

## 4. Discussion

Nine hundred and ninety-nine adolescent lyceum students were recruited in this validation study. To the best of our knowledge, this is the first validation study of the MSPSS in a Greek population of adolescent students aged 15–18. The validated Greek version of the MSPSS holds potential for use in future screening and intervention studies among adolescents. The results of this study demonstrate that the MSPSS has good internal consistency and reliability, with a Cronbach’s alpha ranging from 0.899 to 0.938. There is no evidence of bias in any of the item’s answers, as they all seem to have satisfactory differentiative power and variance.

The MSPSS measures an individual’s perceived social support across different dimensions, such as family, friends, and significant others. The MSPSS is employed in research to explore the relationship between social support and various outcomes, such as mental health (e.g., depression, anxiety), coping with stress, and well-being. It has been used in clinical settings to assess perceived support of social networking and, also, to tailor interventions aimed at the improvement of perceived social support. Several studies had been conducted in different cultural populations and in plenty special populations such as patients with cancer, breast cancer, multiple sclerosis, HIV/AIDS, caregivers of dementia patients, parents of children with cerebral palsy, mothers of children with developmental disabilities, older adults, school teachers, pregnant women, and university students [38,53,54,55,56,57,58,59,60,61,62,63,64]. It has been culturally adapted and validated in the Greek general population [36]. The optimum EFA and CFA conducted during the present study demonstrated that this scale can also be used in older adolescents in Greece, while the initial version was applied to undergraduate students aged 17–22 years, a range close to our target group aged 15–18 years. Most studies with the aim of validation of the MSPSS resulted in the three-factor model as the optimum best fit for the available data. However, some studies have revealed a two-factor model with combined dimensions of ‘Significant Other’ and ‘Friends’, probably due to cultural differences, as it seems the significant others of the adolescents are more likely to be their peers [65]. Similarly, in a study conducted in a sample of Nigerian patients with stroke, and another in Chilean elders, the two-factor model combined the factors of ‘Family’ and ‘Significant Others’, and the two-factor model demonstrated a better fit [66,67]. All items loaded properly in each dimension for the rest of the validation study, with an exception appearing in a sample of Ghanian adolescents, where item 10 was loaded barely stronger on a different subscale (Friends) instead of significant others [68]. In the current research, for the three-factor model, 80.80% of the variance was accounted for. The findings are both consistent with those of other studies in comparable populations, but it appears that higher scores of internal consistency would have been supported [68,69,70,71,72]. One strength of the presented study was the employment of both EFA and CFA to produce unbiased results, where the scores of factor loadings of EFA and CFA were found to be large (above 0.7). EFA and CFA were not assessed in every validation study of the MSPSS. For example, item 7 (“counting on my friends”) has demonstrated low scores of factor loading in the Indonesian and the Ghanian version [68,73].

This study presented high internal consistency in accordance with the high reliability of the initial version in adolescent populations of Indonesian, Ghanaian, Chinese, and Bangladeshi descent [35,69,71,73]. These outcomes were revealed not only in the score of the overall scale, but, also, in the score of each component, separately. The weak points found in the Hong Kong and Mexican American versions where the ‘Significant Other’ dimension had a low Cronbach’s alpha did not appeared in the Greek validation of high school adolescents [70,74]. This study established the MSPSS as highly reliable in detecting gaps in perceived social support, as its scores of internal consistency and reliability were higher when compared with other studies in the Greek population [36,37,38], or even the initial version [35]. The strong internal consistency of the MSPSS has been confirmed by several studies across the world [64,65,66,75,76].

The correlation between ‘Friends’ and ‘Family’ subscales was found to be strong but lower than the scores of the other subscales, implying that these two dimensions seem to be independent enough to be separated. On the other hand, the correlations of the dimensions of ‘Significant Other’ with ‘Friends’ and ‘Family’ showed strong correlation, implying a significant perceived emotional equality. One possible explanation lies in the similar emotional context for a ’Significant other’, or a ’Friend’, or the ‘Parents’.

EFA helps to find patterns in a group of variables, whereas CFA checks if a specific theory about those variables holds true. Both techniques are used to identify the relationships between observed variables and their underlying latent constructs. Of note, the employment of the aforementioned as the confirmation of the three-factor structure constitutes a strength point in the current study. Another strong point is the large sample size. With the exception of the validation study of Hong Kong [74], most validation studies of larger population countries included samples of 200–300 adolescents, while in this study 999 adolescents were included. Thus, the current study enriches the current medical literature by validating the psychometric evaluation of the MSPSS in adolescents, a target group with increasing need of social support. The lack of a criterion-related validity assessment is a significant limitation of the current study. The strength of the validation process is limited by the scale’s lack of comparison with a recognized measure of perceived social support, despite its strong internal consistency and structural validity. In order to strengthen the construct validity and improve the findings’ interpretability and practical relevance, future research is urged to incorporate criterion measures. Future studies could explore longitudinal stability and predictive validity of the Greek MSPSS in this population.

## 5. Conclusions

Screening for gaps in perceived social support of the adolescent population is essential in the practice of Adolescent Medicine. To our knowledge, this is the first Greek version of the MSPSS scale as an instrument for measuring perceived social support in Greek adolescents. In conclusion, we provided the external validation of the MSPSS in the Greek context. The results of this study clearly demonstrated the MSPSS as internally reliable, and the factor structure of the MSPSS seems to be similar to the originally hypothesized three-factor structure. Thus, the Greek version of the MSPSS is a valid instrument that can be used in medical and psychologic practice settings to screen adolescents for gaps in social support.

## Figures and Tables

**Table 1:** Demographic characteristics of the sample (N = 999).

	n	%
Gender identification		
Male	331	33.1
Female	663	66.4
Other Gender	5	0.5
Grades of the upper secondary education school (lyceum)		
First	142	14.2
Second	349	34.9
Third	508	50.9
Type of school		
High school (Lyceum)	902	90.7
Vocational High School	46	4.6
Model High School	18	1.8
Experimental high School	11	1.1
Art High School	17	1.7

Notes. Values are referred to absolute and relative frequencies (%).

**Table 2:** Explanatory Factor Analysis for MSPSS.

Item	Label	Mean ± SD	MSA	Factor Loadings	Cronbach’s Alpha/McDonald’s Omega
Significant Other					0.899/0.899
MSPSS 1	Special person around	5.97 ± 1.41	0.927	0.724	
MSPSS 2	Special person for joys and sorrows	5.96 ± 1.43	0.916	0.765	
MSPSS 5	Special person for comfort to me	5.51 ± 1.64	0.936	0.788	
MSPSS 10	Special person who cares about my feelings	5.98 ± 1.43	0.955	0.724	
Family					0.913/0.915
MSPSS 3	Help from family	5.98 ± 1.40	0.896	0.853	
MSPSS 4	Emotional help and support from my family	5.23 ± 1.76	0.908	0.822	
MSPSS 8	Talking about my problems with my family	4.90 ± 1.90	0.922	0.810	
MSPSS 11	My family helps me make decisions	5.71 ± 1.51	0.904	0.856	
Friends					0.937/0.938
MSPSS 6	Help from friends	5.25 ± 1.53	0.913	0.872	
MSPSS 7	Counting on my friends	5.03 ± 1.71	0.902	0.886	
MSPSS 9	Friends for joys and sorrows	5.63 ± 1.59	0.922	0.857	
MSPSS 12	Talking to friends about my problems	5.41 ± 1.65	0.940	0.849	

Notes. Abbreviations—MSPSS: Multidimensional Scale of Perceived Social Support, SD: standard deviation, MSA: Measures of Sampling Adequacy. Factor Loadings were extracted after rotation with the Varimax method.

## Data Availability

The data presented in this study are available on request from the corresponding author.

## Abbreviations

The following abbreviations are used in this manuscript:

SSQ	Social Support Questionnaire
ISEL	Interpersonal Support Evaluation List
SSI	Social Support Inventory
CASSS	Child and Adolescent Social Support Scale
MSPSS	Multidimensional Scale of Perceived Social Support
ASPETE	School of Pedagogical and Technological Education
EFA	Exploratory Factor Analysis
CFA	Confirmatory Factor Analysis
CFI	Comparative Fit Index
TLI	Tucker–Lewis Index
RMSEA	Root Mean Square Error of Approximation
CI	Confidence Interval

## References

[1] Stromajer G.P., Csima M., Ivancsik R., Varga B., Takacs K., Stromajer-Racz T. (2023). Stress and Anxiety among High School Adolescents: Correlations between Physiological and Psychological Indicators in a Longitudinal Follow-Up Study. Children.

[2] Gülaçtı F. (2010). The effect of perceived social support on subjective well-being. Procedia—Soc. Behav. Sci..

[3] Ghafari R., Mirghafourvand M., Rouhi M., Osouli Tabrizi S. (2021). Mental health and its relationship with social support in Iranian students during the COVID-19 pandemic. BMC Psychol..

[4] Ruiz J., Prather C.C., Kauffman E.E., Gellman M.D., Turner J.R. (2013). Social Support. Encyclopedia of Behavioral Medicine.

[5] Gautam S., Jain A., Chaudhary J., Gautam M., Gaur M., Grover S. (2024). Concept of mental health and mental well-being, it’s determinants and coping strategies. Indian J. Psychiatry.

[6] Acoba E.F. (2024). Social support and mental health: The mediating role of perceived stress. Front. Psychol..

[7] Ozbay F., Johnson D.C., Dimoulas E., Morgan C.A., Charney D., Southwick S. (2007). Social support and resilience to stress: From neurobiology to clinical practice. Psychiatry.

[8] Drageset J., Haugan G., Eriksson M. (2021). Social Support. Health Promotion in Health Care—Vital Theories and Research.

[9] Reblin M., Uchino B.N. (2008). Social and emotional support and its implication for health. Curr. Opin. Psychiatry.

[10] Wu J.J., Khan H.A., Chien S.H., Lee Y.P. (2019). Impact of Emotional Support, Informational Support, and Norms of Reciprocity on Trust Toward the Medical Aesthetic Community: The Moderating Effect of Core Self-Evaluations. Interact. J. Med. Res..

[11] Schultz B.E., Corbett C.F., Hughes R.G. (2022). Instrumental support: A conceptual analysis. Nurs. Forum.

[12] Cross C.J., Nguyen A.W., Chatters L.M., Taylor R.J. (2018). Instrumental Social Support Exchanges in African American Extended Families. J. Fam. Issues.

[13] Liu Q., Jiang M., Li S., Yang Y. (2021). Social support, resilience, and self-esteem protect against common mental health problems in early adolescence: A nonrecursive analysis from a two-year longitudinal study. Medicine.

[14] Zhang C. (2019). Family Support or Social Support? The Role of Clan Culture. J Popul Eco.

[15] Bardi A., Guerra V. (2011). Cultural Values Predict Coping Using Culture as an Individual Difference Variable in Multicultural Samples. J. Cross-Cult. Psychol..

[16] Luong G., Arredondo C.M., Charles S.T. (2020). Cultural differences in coping with interpersonal tensions lead to divergent shorter- and longer-term affective consequences. Cogn. Emot..

[17] McGuire A.P., Gauthier J.M., Anderson L.M., Hollingsworth D.W., Tracy M., Galea S., Coffey S.F. (2018). Social Support Moderates Effects of Natural Disaster Exposure on Depression and Posttraumatic Stress Disorder Symptoms: Effects for Displaced and Nondisplaced Residents. J. Trauma. Stress..

[18] Makwana N. (2019). Disaster and its impact on mental health: A narrative review. J. Fam. Med. Prim. Care.

[19] Choi K.W., Lee Y.H., Liu Z., Fatori D., Bauermeister J.R., Luh R.A., Clark C.R., Brunoni A.R., Bauermeister S., Smoller J.W. (2023). Social support and depression during a global crisis. Nat. Ment. Health.

[20] Brailovskaia J., Margraf J. (2022). Positive mental health and mindfulness as protective factors against addictive social media use during the COVID-19 outbreak. PLoS ONE.

[21] Wickramaratne P.J., Yangchen T., Lepow L., Patra B.G., Glicksburg B., Talati A., Adekkanattu P., Ryu E., Biernacka J.M., Charney A. (2022). Social connectedness as a determinant of mental health: A scoping review. PLoS ONE.

[22] Asimopoulos C., Teloni D.D. (2017). Social work and the psychosocial effects of the economic crisis in Greece: Challenges for new radical directions in services, theory and values. Comunitania. Rev. Int. De Trab. Soc. Y Cienc. Soc..

[23] Giotakos O., Karabelas D., Kafkas A. (2011). Financial crisis and mental health in Greece. Psychiatr. = Psychiatr..

[24] Koutsogeorgopoulou V., Matsaganis M., Leventi C., Schneider J.-D. (2014). Fairly Sharing the Social Impact of the Crisis in Greece.

[25] Wei P. (2022). The impact of social support on students’ mental health: A new perspective based on fine art majors. Front. Psychol..

[26] Jieyi H., Kiu C.C., Baojian X. (2022). How academic performance influences social integration: The moderation effect of cultural distance among Chinese cross-borderers. Brain Behav..

[27] Dupont S., Galand B., Nils F. (2015). The impact of different sources of social support on academic performance: Intervening factors and mediated pathways in the case of master’s thesis. Rev. Eur. De Psychol. Appliquée/Eur. Rev. Appl. Psychol..

[28] Kafetsios K. (2006). Social Support and Well-Being in Contemporary Greek Society: Examination of Multiple Indicators at Different Levels of Analysis. Soc. Indic. Res..

[29] Kafetsios K. (2007). Gender, social support, and well-being: Evidence from a Greek community sample. Interpersona An. Int. J. Pers. Relatsh..

[30] Sarason I.G., Levine H.M., Basham R.B., Sarason B.R. (1983). Assessing social support: The Social Support Questionnaire. J. Personal. Soc. Psychol..

[31] Cohen S., Hoberman H.M. (1983). Positive Events and Social Supports as Buffers of Life Change Stress. J. Appl. Soc. Psychol..

[32] Delistamati E., Samakouri M.A., Davis E.A., Vorvolakos T., Xenitidis K., Livaditis M. (2006). Interpersonal Support Evaluation List (ISEL)--college version: Validation and application in a Greek sample. Int. J. Soc. Psychiatry.

[33] Timmerman I.G.H., Emanuels-Zuurveen E.S., Emmelkamp P.M.G. (2000). The Social Support Inventory (SSI): A brief scale to assess perceived adequacy of social support. Clin. Psychol. Psychother..

[34] Malecki C., Demaray M. (2002). Measuring perceived social support: Development of the child and adolescent social support scale (CASSS). Psychol. Sch..

[35] Zimet G.D., Dahlem N.W., Zimet S.G., Farley G.K. (1988). The Multidimensional Scale of Perceived Social Support. J. Personal. Assess..

[36] Katsiroumpa A., Moisoglou I., Konstantakopoulou O., Vraka I., Gallos P., Tsiachri M., Tsakalaki A., Galanis P. (2023). Translation and Validation of the “Multidimensional Scale of Perceived Social Support” in the Greek General Population.

[37] Tsilika E., Galanos A., Polykandriotis T., Parpa E., Mystakidou K. (2019). Psychometric Properties of the Multidimensional Scale of Perceived Social Support in Greek Nurses. Can. J. Nurs. Res..

[38] Theofilou P. (2015). Translation and Cultural Adaptation of the Multidimensional Scale of Perceived Social Support for Greece. Health Psychol. Res..

[39] Comrey A.L., Lee H.B. (1992). A First Course in Factor Analysis.

[40] Costello A.B., Osborne J. (2005). Best Practices in Exploratory Factor Analysis: Four Recommendations for Getting the Most From Your Analysis. Pract. Assess. Res. Eval..

[41] Khan M.N., Dherani M., Chiumento A., Atif N., Bristow K., Sikander S., Rahman A. (2017). Evaluating feasibility and acceptability of a local psycho-educational intervention for pregnant women with common mental problems affected by armed conflict in Swat, Pakistan: A parallel randomized controlled feasibility trial. Int. J. Soc. Psychiatry.

[42] Solano-Flores G., Backhoff E., Contreras-Niño L.Á. (2009). Theory of test translation error. Int. J. Test..

[43] van de Vijver F., Hambleton R.K. (1996). Translating tests: Some practical guidelines. Eur. Psychol..

[44] R Core Team (2020). R: A Language and Environment for Statistical Computing.

[45] Rosseel Y. (2012). lavaan: An R Package for Structural Equation Modeling. J. Stat. Softw..

[46] Lishinski A. (2024). lavaanPlot: Path Diagrams for ‘Lavaan’ Models via ‘DiagrammeR’. R Package Version 0.8.1. https://lavaanplot.alexlishinski.com.

[47] Cronbach L.J. (1951). Coefficient alpha and the internal structure of tests. Psychometrika.

[48] Kaiser H.F., Rice J. (1974). Little Jiffy, Mark Iv. Educ. Psychol. Meas..

[49] Hooper D., Coughlan J., Mullen M. (2007). Structural Equation Modeling: Guidelines for Determining Model Fit. Electron. J. Bus. Res. Methods.

[50] Hair J.F., Black W.C., Babin B.J., Anderson R.E. (2010). Multivariate Data Analysis..

[51] Hu L.t., Bentler P.M. (1999). Cutoff criteria for fit indexes in covariance structure analysis: Conventional criteria versus new alternatives. Struct. Equ. Model. A Multidiscip. J..

[52] West S.G., Taylor A.B., Wu W. (2012). Model Fit and Model Selection in Structural Equation Modeling. Handb. Struct. Equ. Model..

[53] Song W., Mansor N.S., Shari N.I., Azman N., Zhang R., Leong Bin Abdullah M.F.I. (2023). Validation of the Malay version of the Multidimensional Scale of Perceived Social Support (MSPSS-M) among patients with cancer in Malaysia. PLoS ONE.

[54] Perez-Villalobos C., Briede-Westermeyer J.C., Schilling-Norman M.J., Contreras-Espinoza S. (2021). Multidimensional scale of perceived social support: Evidence of validity and reliability in a Chilean adaptation for older adults. BMC Geriatr..

[55] Wang Y., Wan Q., Huang Z., Huang L., Kong F. (2017). Psychometric Properties of Multi-Dimensional Scale of Perceived Social Support in Chinese Parents of Children with Cerebral Palsy. Front. Psychol..

[56] Kim M., Yeom H.E., Jung M.S. (2022). Validation and psychometric properties of the multidimensional scale of perceived social support among Korean breast cancer survivors. Asia Pac. J. Oncol. Nurs..

[57] Ebrahim M.T., Alothman A.A. (2022). The reliability and validity of the multidimensional scale of perceived social support (MSPSS) in mothers of children with developmental disabilities in Saudi Arabia. Res. Autism Spectr. Disord..

[58] Cartwright A.V., Pione R.D., Stoner C.R., Spector A. (2022). Validation of the multidimensional scale of perceived social support (MSPSS) for family caregivers of people with dementia. Aging Ment. Health.

[59] Kieu P.T., Vuong N.L., Dung D.V. (2023). Validation of Multidimensional Scale of Perceived Social Support (MSPSS) in Vietnamese Among People Living with HIV/AIDS. AIDS Behav..

[60] Sharif M., Zaidi A., Waqas A., Malik A., Hagaman A., Maselko J., LeMasters K., Liaqat R., Bilal S., Bibi T. (2021). Psychometric Validation of the Multidimensional Scale of Perceived Social Support During Pregnancy in Rural Pakistan. Front. Psychol..

[61] Calderon C., Ferrando P.J., Lorenzo-Seva U., Gomez-Sanchez D., Fernandez-Montes A., Palacin-Lois M., Antonanzas-Basa M., Rogado J., Manzano-Fernandez A., Ferreira E. (2021). Multidimensional Scale of Perceived Social Support (MSPSS) in cancer patients: Psychometric properties and measurement invariance. Psicothema.

[62] Ho S.K., Chan E.S. (2017). Modification and validation of the multidimensional scale of perceived social support for Chinese school teachers. Cogent Educ..

[63] Brugnoli A.V.M., Goncalves T.R., Silva R., Pattussi M.P. (2022). Evidence of the validity of the Multidimensional Scale of Perceived Social Support (MSPSS) in university students. Cien Saude Colet..

[64] Adamczyk K. (2013). Development and validation Of the Polish-language version of the Multidimensional Scale of Perceived Social Support (MSPSS). Rev. Int. De Psychol. Soc..

[65] Chou K.-L. (2000). Assessing Chinese adolescents’ social support: The multidimensional scale of perceived social support. Personal. Individ. Differ..

[66] Mohammad A.H., Al Sadat N., Loh S.Y., Chinna K. (2015). Validity and reliability of the hausa version of multidimensional scale of perceived social support index. Iran. Red Crescent Med. J..

[67] Arechabala Mantuliz M.C., Miranda Castillo C. (2002). Validacion de una escala de apoyo social percibido en un grupo de adultos mayores adscritos a un programa de hipertension de la region metropolitana. Cienc. Y Enfermería.

[68] Canty-Mitchell J., Zimet G.D. (2000). Psychometric properties of the Multidimensional Scale of Perceived Social Support in urban adolescents. Am. J. Community Psychol..

[69] Wilson A., Yendork J.S., Somhlaba N.Z. (2017). Psychometric Properties of Multidimensional Scale of Perceived Social Support among Ghanaian Adolescents. Child Indic. Res..

[70] Edwards L.M. (2004). Measuring Perceived Social Support in Mexican American Youth: Psychometric Properties of the Multidimensional Scale of Perceived Social Support. Hisp. J. Behav. Sci..

[71] Murshid M.E., Chen S., Rahman M.M., Islam M.Z., Shimpuku Y., Rahman Era N., Kumar S., Haque M. (2023). Reliability and Validity of the Multidimensional Scale of Perceived Social Support Among Women and Adolescent Girls With Disabilities in Selected Sub-districts of Bangladesh. Cureus.

[72] Trejos-Herrera A.M., Bahamón M.J., Alarcón-Vásquez Y., Vélez J.I., Vinaccia S. (2018). Validity and reliability of the Multidimensional Scale of Perceived Social Support in Colombian adolescents. Psychosoc. Interv..

[73] Laksmita O.D., Chung M.H., Liao Y.M., Chang P.C. (2020). Multidimensional Scale of Perceived Social Support in Indonesian adolescent disaster survivors: A psychometric evaluation. PLoS ONE.

[74] Cheng S.-T., Chan A. (2004). The Multidimensional Scale of Perceived Social Support: Dimensionality and age and gender differences in adolescents. Personal. Individ. Differ..

[75] Stewart R.C., Umar E., Tomenson B., Creed F. (2014). Validation of the multi-dimensional scale of perceived social support (MSPSS) and the relationship between social support, intimate partner violence and antenatal depression in Malawi. BMC Psychiatry.

[76] Park H., Nguyen T., Park H. (2012). Validation of multidimensional scale of perceived social support in middle-aged Korean women with diabetes. Asia Pac. J. Soc. Work Dev..

